# Proper Actin Ring Formation and Septum Constriction Requires Coordinated Regulation of SIN and MOR Pathways through the Germinal Centre Kinase MST-1

**DOI:** 10.1371/journal.pgen.1004306

**Published:** 2014-04-24

**Authors:** Yvonne Heilig, Anne Dettmann, Rosa R. Mouriño-Pérez, Kerstin Schmitt, Oliver Valerius, Stephan Seiler

**Affiliations:** 1 Institute for Biology II – Molecular Plant Physiology, Albert-Ludwigs University Freiburg, Freiburg, Germany; 2 Department of Microbiology, Center for Scientific Research and Higher Education of Ensenada (CICESE), Baja California, Mexico; 3 Institute for Microbiology and Genetics, University of Goettingen, Goettingen, Germany; 4 Freiburg Institute for Advanced Studies (FRIAS), Albert-Ludwigs University Freiburg, Freiburg, Germany; University College Dublin, Ireland

## Abstract

Nuclear DBF2p-related (NDR) kinases constitute a functionally conserved protein family of eukaryotic regulators that control cell division and polarity. In fungi, they function as effector kinases of the morphogenesis (MOR) and septation initiation (SIN) networks and are activated by pathway-specific germinal centre (GC) kinases. We characterized a third GC kinase, MST-1, that connects both kinase cascades. Genetic and biochemical interactions with SIN components and life cell imaging identify MST-1 as SIN-associated kinase that functions in parallel with the GC kinase SID-1 to activate the SIN-effector kinase DBF-2. SID-1 and MST-1 are both regulated by the upstream SIN kinase CDC-7, yet in an opposite manner. Aberrant cortical actomyosin rings are formed in Δ*mst-1*, which resulted in mis-positioned septa and irregular spirals, indicating that MST-1-dependent regulation of the SIN is required for proper formation and constriction of the septal actomyosin ring. However, MST-1 also interacts with several components of the MOR network and modulates MOR activity at multiple levels. MST-1 functions as promiscuous enzyme and also activates the MOR effector kinase COT-1 through hydrophobic motif phosphorylation. In addition, MST-1 physically interacts with the MOR kinase POD-6, and dimerization of both proteins inactivates the GC kinase hetero-complex. These data specify an antagonistic relationship between the SIN and MOR during septum formation in the filamentous ascomycete model *Neurospora crassa* that is, at least in part, coordinated through the GC kinase MST-1. The similarity of the SIN and MOR pathways to the animal Hippo and Ndr pathways, respectively, suggests that intensive cross-communication between distinct NDR kinase modules may also be relevant for the homologous NDR kinases of higher eukaryotes.

## Author Summary

Cytokinesis is a fundamental cellular process essential for cell proliferation of uni- and multicellular organisms. The molecular pathways that regulate cytokinesis are highly complex and involve a large number of components that form elaborate interactive networks. The fungal septation initiation network (SIN) functions as tripartite kinase cascade that connects cell cycle progression with the control of cell division. Mis-regulation of the homologous Hippo pathway in animals results in excessive proliferation and formation of tumors, underscoring the conservation and importance of these kinase networks. A second morphogenesis (MOR) pathway involves homologous components and is controlling cell polarity in fungi and higher eukaryotes. Here we show that the promiscuous functioning Ste20-related kinase MST-1 has a dual role in regulating SIN and MOR network function. Moreover, SIN and MOR coordination through MST-1 can be achieved in an enzyme-independent manner through hetero-dimerization of germinal centre kinases, providing an additional level for activity regulation of signaling networks that is not dependent on phosphate transfer. Given the functional conservation of NDR kinase signaling modules and their regulation, our work may define general mechanisms by which NDR kinase pathway are coordinated in fungi and higher eukaryotes.

## Introduction

Coordination of cell growth and division is a fundamental subject in biology. Successful cytokinesis relies on the coordinated assembly and activation of an actomyosin-based contractile ring, which must be regulated in a spatially and temporally precise manner [1]–[3]. The underlying molecular pathways are highly complex and involve a large number of components forming elaborate interactive networks. Central components of these networks are the highly conserved nuclear Dbf2p-related (NDR) kinases, which are important for morphogenesis and proliferation in all eukaryotes analyzed to date [4]–[6]. These kinases represent two functional subgroups in fungi and animals. Dbf2p subfamily members are effector kinases of the fungal septation initiation network (SIN; homologous to the animal Hippo pathway; [7]), which consists of a cascade of three kinases that connects cell cycle progression with the initiation of cytokinesis [8]. Activation of the SIN by a spindle pole body (SPB)-associated small GTPase activates a Cdc14p-like phosphatase, which triggers mitotic exit [9]. Moreover, activated Dbf2p relocates to the future site of septum formation, where the kinase is required for the assembly and constriction of the cortical actomyosin ring (CAR; [10], [11]). The second group of NDR kinases controls polarity as components of a homologous morphogenesis network (the fungal MOR network; homologous to the animal Ndr1/2 pathway) [5]. In fungi this pathway plays a critical role in the polar organization of the actin cytoskeleton at cell ends, which seems, at least in part, to be achieved through regulation of Rho-type GTPases [12]–[15]. In addition, the MOR regulates the expression of multiple genes with cell wall-related functions in unicellular yeasts, including genes required for efficient cell separation [16]–[18].

Multiple data indicate that the SIN and MOR function in concert in complex and mutual antagonistic manner to coordinate the remodeling of the actin cytoskeleton during cell growth and division [5], [10], [11]. Inhibition of the fission yeast MOR network by the SIN has been shown to promote cytokinesis, while polar growth in interphase requires inhibition of the SIN by the MOR [19]–[21]. Moreover, the activity of the MOR is SIN-dependent [22], [23], consistent with the hypothesis of the MOR acting as SIN effector after septum constriction to allow cell separation. Significantly, the animal Hippo and Ndr1/2 kinase pathways also have opposing effects on cell morphogenesis and proliferation [24]–[26], indicating that this interdependence of the two NDR kinase modules is conserved among eukaryotes.

In contrast to the total separation of mother and daughter cells, which is characteristic for unicellular yeasts, the vast majority of fungi form mycelial colonies that consist of networks of branched hyphae and are compartmentalized by incomplete septa. The septal pores enable communication and transport of cytoplasm and organelles between adjacent cells. Despite the importance of septation for growth, proliferation and differentiation of molds, our understanding of septum formation and its regulation in filamentous fungi is highly fragmentary [27]–[29]. Genetic and biochemical analysis in the model molds *Neurospora crassa* and *Aspergillus nidulans* suggest that cell cycle-dependent signals of a subset of competent mitotic nuclei activate the SIN [30]–[32]. This, in turn, is critical for the cortical localization of a RHO-4/BUD-3 GTPase module complexed by its anillin-type scaffold BUD-4, which allows the generation of the CAR through a second RHO-4/RGF-3 GTPase complex [33]–[35]. In addition to these positive regulators of septum formation, several negative regulators were identified in *N. crassa*, whose mutation result in hyperseptated strains [36]. Most notably are LRG-1, a RHO-1-specific GTPase activating protein [14] and COT-1, POD-6 and two MOB-2 proteins, the central elements of the *N. crassa* MOR network [37]–[39]. Moreover, components of the RHO-1 module and the MOR network localize to forming septa, providing additional support for a function of these proteins during septation [14], [40]–[42].

We have recently determined that a hierarchical, tripartite SIN cascade, consisting of CDC-7, SID-1 and DBF-2, together with their regulatory subunits CDC-14 and MOB-1, respectively, are the central components of the SIN and are essential for septum formation in *N. crassa* [39], [43]. However, the comparative phenotypic characterization of mutants in these SIN components revealed that Δ*sid-1* and Δ*cdc-14* deletion strains behave differently than mutants in the remaining SIN components, suggesting that the presentation of the SIN as a linear kinase cascade may represent a simplified view. In this study, we characterized MST-1, the third germinal centre (GC) kinase present in *N. crassa* and other fungi, which we show is important for proper CAR formation and septum constriction. We provide evidence that MST-1 functions in parallel to SID-1 as part of the SIN, but also that MST-1 has multiple functions in regulating the MOR. Consequently, MST-1 plays an important role in coordinating the antagonistic functions of the SIN and MOR pathways during septum formation.

## Results

### The germinal centre kinase MST-1 displays features reminiscent of SIN as well as MOR components

**Figure 1:**
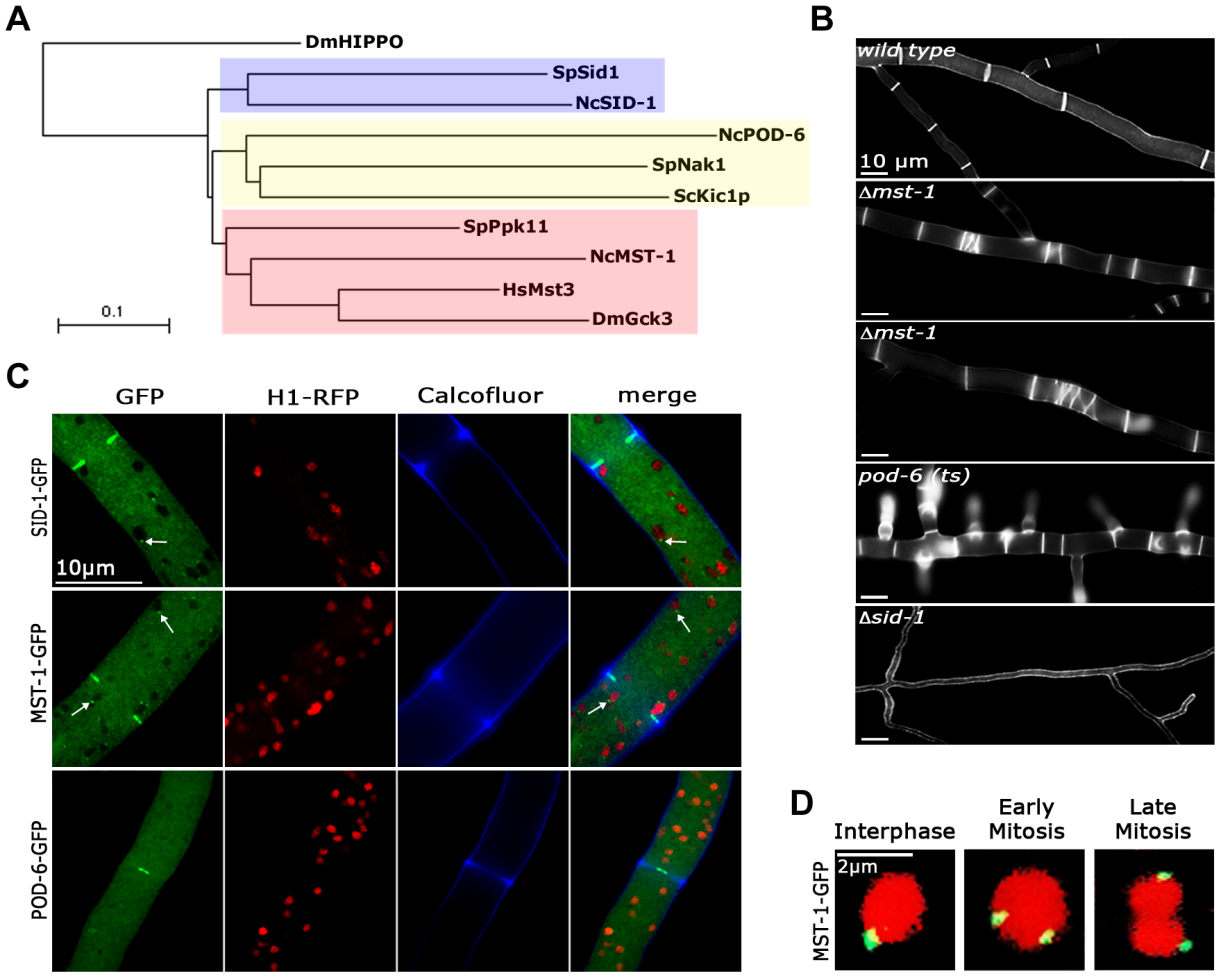
MST-1 displays features reminiscent of SIN as well as MOR components. (**A**) Phylogenetic comparison of fungal GC kinases. The tree was generated by the neighbour-joining method based on a ClustalW alignment of the indicated *S. cerevisiae, S. pombe and N. crassa* proteins. HsMst3 and DmGck3 were used as examples for animal group III GC kinases. The GC kinase II DmHippo was used as outgroup member (multiple alignment parameters: open gap penalty 10.0, extend gap penalty 0.0, delay divergent 40%, gap distance 8, similarity matrix blosum). An extended phylogram of fungal GC kinases is found in Figure S1. (**B**) Phenotypic characteristics of Δ*mst-1*, *pod-6(ts) and* Δ*sid-1* during septum formation. Note the presence of closely spaced septa (second panel) and abnormal spirals (third panel) in Δ*mst-1*. For comparison, *pod-6*-defective cells produce multiple, closely spaced septa, while Δ*sid-1* cells are aseptate. Cell wall and septa were labeled with Calcofluor White. (**C**) Functional GFP fusion proteins of the three GC kinases SID-1, MST-1 and POD-6 localize as constricting rings at septa. SID-1 and MST-1, but not POD-6 also localize to spindle pole bodies (arrows), while POD-6 accumulated as dot in the distal region of the *Spitzenkörper* and as apex-associated crescent (Figure S2). Nuclei were labeled with histone H1-RFP and the cell wall was stained with Calcofluor White. (**D**) MST-1-GFP associated with SPBs of interphase nuclei as well as during early and late mitotic stages (as indicated by nuclear morphology). Nuclei were labeled with histone H1-RFP.

Germinal centre (GC) kinases represent a large family of functionally diverse proteins that can be grouped into eight subfamilies [44]. Fungal GC kinases represent three functionally distinct subgroups of subfamily III (Figure 1 A; Figure S1). *N. crassa* POD-6 and the related budding and fission yeast kinases Kic1p and Nak1, respectively, clustered together, in line with their conserved function as upstream components of the MOR network [38], [45], [46]. The second subgroup is represented by the SIN kinases Sid1/SID-1 [43], [47]. Proteins of the third subgroup are most closely related to animal group III GC kinases. The fission yeast member Ppk11 was recently characterized as auxiliary factor of the MOR pathway that supports cell separation [48], while the *N. crassa* protein NCU00772/MST-1 had been implicated as part of the SIN in a preliminary analysis [49].

**Figure 2:**
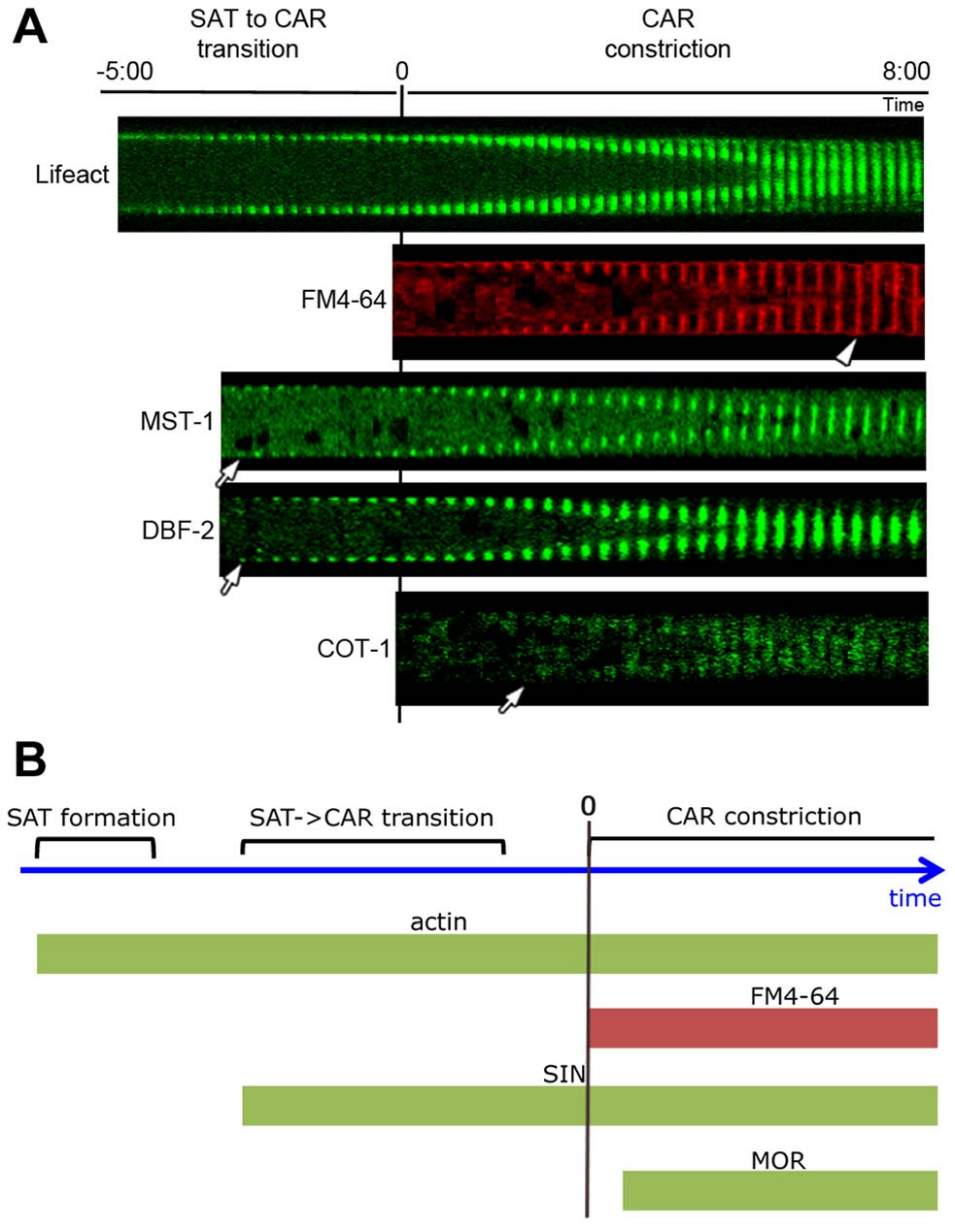
Kinetics of cortex association of SIN and MOR components DBF-2, MST-1 and COT-1 during septum formation. (**A**) Composite images of time series of the indicated proteins during septum formation. The start of plasma membrane invagination was monitored with FM4-64 and defined as time point 0:00 min. Arrows indicate the time point when the GFP-fusion proteins occurred first at the cell cortex and arrowhead the time point of complete CAR constriction. (**B**) Schematic representation of the chronology of emergence of the SIN and MOR at the cell cortex relative to the actin cytoskeleton.

In order to address these discrepancies, we compared localization pattern and mutant characteristics of MST-1/Δ*mst-1* with those of SIN and MOR components. Δ*mst-1* formed multiple, closely spaced septa similar to the hyperseptation defects observed in *MOR* mutants and produced abnormal cross walls in the form of cortical spirals in older hyphal segments (Figure 1 B). However, a MST-1-GFP fusion construct, which complemented all Δ*mst-1* defects, associated with spindle pole bodies (SPBs) and constricting septa (Figure 1 C; Video S1), a localization pattern characteristic for fungal SIN components. Moreover, the association of MST-1 with both SPBs was constitutive and cell cycle independent (Figure 1 D), consistent with the localization of the three *N. crassa* SIN kinases [43]. In contrast, a POD-6-GFP fusion protein localized at the hyphal tip in a dot-like structure in the *Spitzenkörper*, as membrane-associated apical crescent and around the septal pore (Figure 1 C; Figure S2), reflecting the localization pattern of other MOR components [41], [42]. Neither POD-6 nor its target kinase COT-1 and regulatory subunit MOB-2A were detected at SPBs. Therefore, we compared the kinetics of cortex recruitment of the two NDR kinases with that of MST-1 during septum initiation (Figure 2). DBF-2 and MST-1 formed cortex-associated rings 2:50±0:45 min (n = 17) and 2:55±1:25 min (n = 19), respectively, prior to the start of septum constriction, which was monitored by plasma membrane invagination and defined as time point 0:00 min (labeled with FM4-64). In contrast, COT-1 was only observed after CAR constriction had started (0:39±0:13 min; n = 15). Furthermore, the fusion protein strongly labeled the mature septal pore [41], [42].

In addition to the observed septation defects in subapical hyphal regions, Δ*mst-1* displayed only minor vegetative abnormalities. Mycelial extension rates, colony behavior and conidiation pattern were similar to wild type (wt; data not shown). However, sexual differentiation was affected in Δ*mst-1*. Mutant ×wt crosses resulted in ca. 50% of round (yet fully viable) ascospores, in contrast to the typical pea-shaped progeny generated in wt×wt crosses (Figure S3). Moreover, Δ*mst-1*×Δ*mst-1* crosses resulted in the formation of empty perithecia lacking asci, and the formation of ascospores was abolished. We also observed synthetic behavior of Δ*mst-1* with *SIN-*, but not *MOR*-defective strains during sexual development. Δ*mst-1*×Δ*cot-1* crosses resulted in the expected segregation of round and normally shaped ascospores, although the total number of generated ascospores was reduced. In contrast, Δ*mst-1*×Δ*dbf-2* crosses generated empty perithecia and no ascospores. The same synthetic effect was observed in crosses of Δ*mst-1* with Δ*sid-1* or Δ*cdc-7*, while crosses of Δ*mst-1*×Δ*pod-6* produced round as well as pea-shaped spores.

### MST-1 controls proper CAR formation

**Figure 3:**
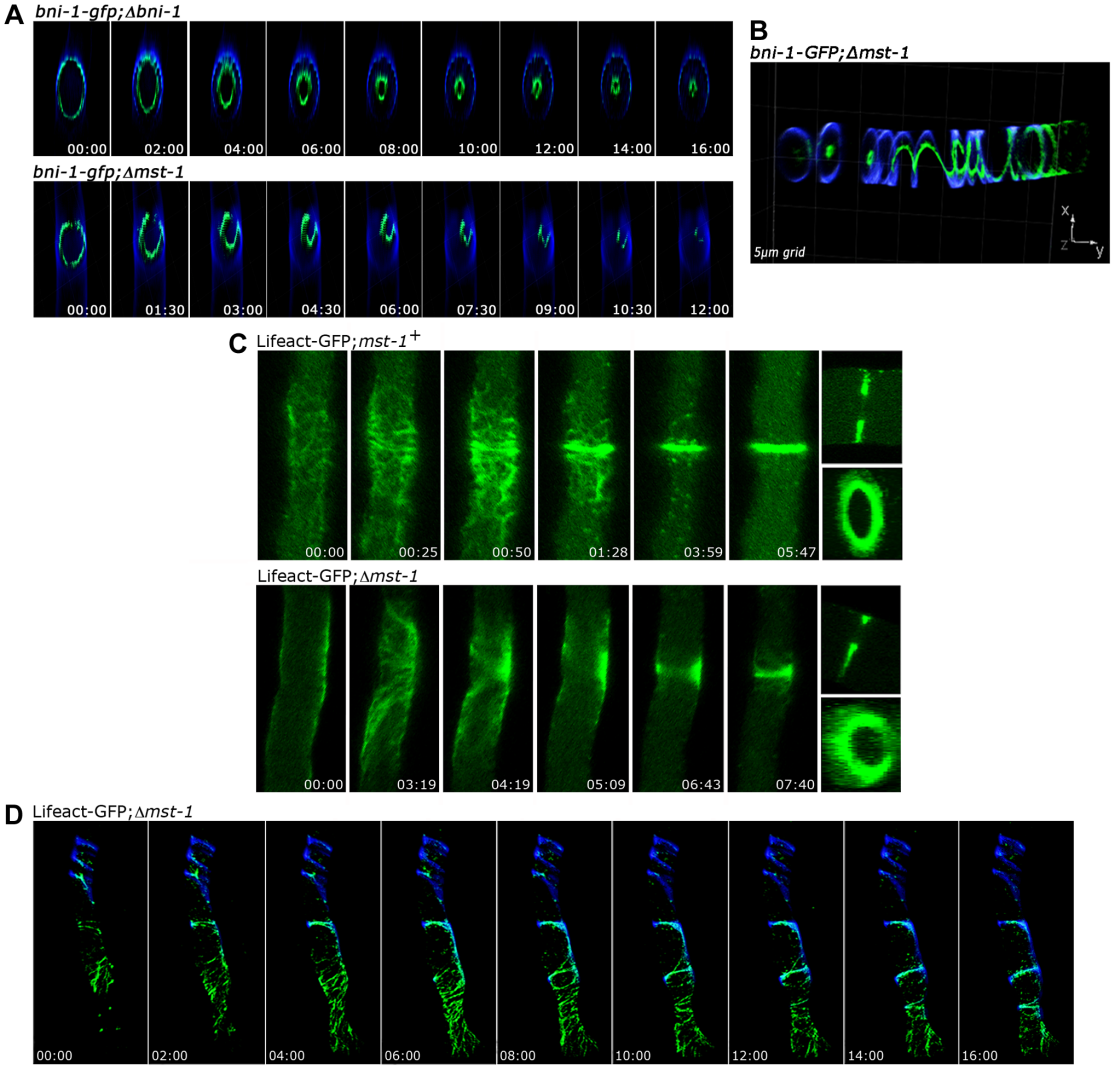
MST-1 is required for proper contractile actin ring formation. (**A**) 4D reconstruction of z-stacks in time lapse series revealed cortical, concentrically constricting BNI-1-GFP rings in wt cells, which resulted in centrally positioned septal pores. In contrast, BNI-1 formed asymmetric and frequently open BNI-1-GFP rings in Δ*mst-*1 that led to acentric CAR constriction and asymmetric septal pores. (**B**) 3D reconstruction of z-stacks illustrates BNI-1-GFP association with extensive cortical Calcofluor White-labeled spirals in Δ*mst-*1. (**C**) Comparison of actin dynamics during CAR assembly and constriction in wt and Δ*mst-*1. Lifeact-GFP labeled a dynamic meshwork of actin cables and patches around the future septation site in wt cells, which subsequently coalesced to form the CAR. The actin meshwork was mis-organized and irregularly distributed in Δ*mst-1* (**D**) 4D reconstruction of z-stacks in time lapse series visualized open actin spirals labeled by Lifeact-GFP, which were unable to constrict. Cell wall, septa and cortical spirals were labeled by Calcofluor White.

Fungal septum formation is driven through the constriction of a cortex-associated actomyosin ring (CAR; [50], [51]). Thus, we compared the dynamics of septum formation in wt and Δ*mst-1* by monitoring the behavior of the sole formin BNI-1 present in *N. crassa* [35]. A functional BNI-1-GFP fusion construct formed cortical rings with uniform signal intensity in wt cells, and septum constriction was concentric, resulting in centrally positioned septal pores (Figure 3 A upper panel; Video S2). In contrast, BNI-1 frequently (i.e. in 38 of 47 septation events analyzed) formed asymmetric rings that led to acentric constriction and asymmetric septal pores in Δ*mst-1* (Figure 3 A lower panel; Videos S3, S4). BNI-1 also associated with extensive cortical Calcofluor White-labeled spirals (Figure 3 B), indicating that the secretory machinery is still correctly targeted and that growth of the cell wall at these sites is not inhibited through the mis-organized formin rings and spirals. Moreover, CAR assembly and constriction was directly monitored using a Lifeact-GFP construct recently developed for *N. crassa* [52]. Lifeact-GFP labeled a mesh of F-actin cables and patches named the septal actin tangle (SAT), which appeared approximately >5 min prior to septum constriction at the future septation site in wt (Figure 2; [52], [53]). After 2–3 min, the SAT coalesced to form the CAR (Figure 3 C upper panel; Video S5). In contrast to wt, the F-actin meshwork was mis-organized and the actin cables were irregularly distributed in Δ*mst-1*, and the SAT to CAR transition lasted much longer (8:30±2:00 min in Δ*mst-1* (n = 12) compared to 2:30±0:30 min in wt (n = 15)). This failure of correct CAR assembly resulted in acentric constriction and asymmetric position of septal pores or the formation of open actin spirals, which were unable to constrict (Figure 3 C lower panel, Figure 3 D; Videos S6, S7).

### The SIN kinase CDC-7 regulates SID-1 and MST-1 in an antagonistic manner

**Figure 4:**
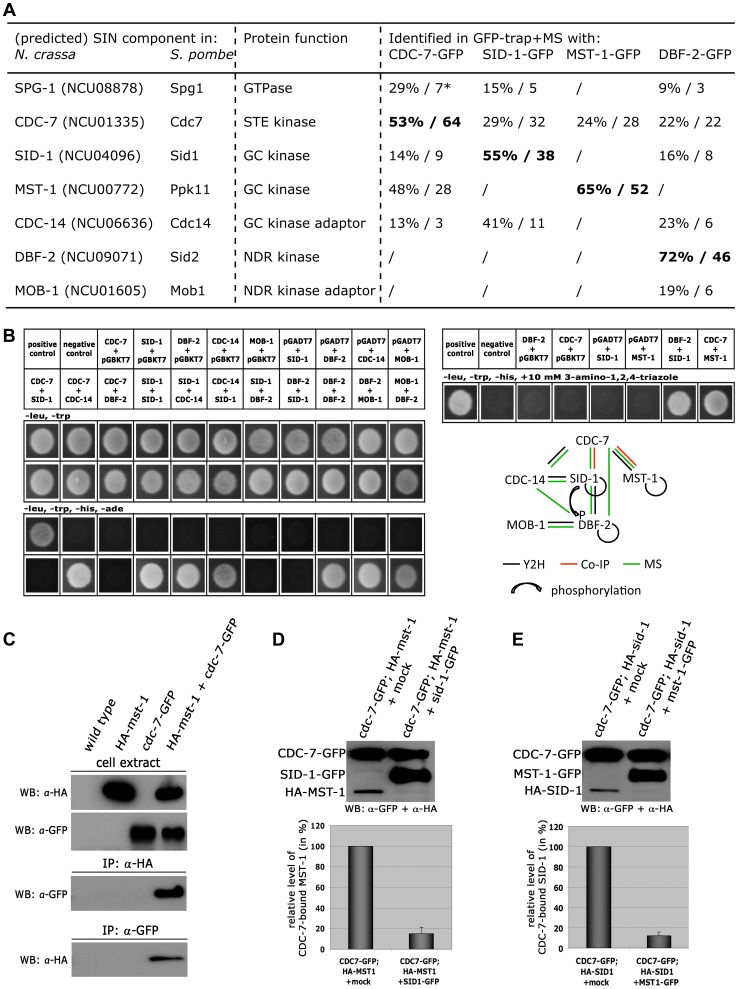
CDC-7 forms mutually exclusive complexes with SID-1 and MST-1. (**A**) AP-MS data from two independent biological replicates were used for identification of predicted SIN components. * denotes % protein coverage and number of unique peptides of the identified protein. Only SIN components identified in both replicate purifications and absent from a control data set obtained in purifications with GFP-expressing cells are displayed (Table S1). GFP-fusion constructs used as bait proteins are in bold type. (**B**) Yeast two-hybrid tests with the indicated SIN constructs and summary schema of the observed interactions. (**C**) Reciprocal co-immunoprecipitation experiments of CDC-7-GFP and HA-MST-1 from cell extracts co-expressing both functionally tagged proteins indicate interaction of the two kinases. (**D, E**) GC-kinase displacement assays of precipitated CDC-7-GFP/GC-kinase complexes. Addition of a second, separately purified GC kinase, but not a mock-IP precipitate to these complexes reduced the abundance of the co-purified GC kinase. The upper panel displays a representative experiment, while three independent experiments are quantified in the lower graph.

GFP-trap affinity purification experiments coupled to mass spectrometry (MS) allowed determining MST-1-interacting proteins and its relationship to the SIN (Figure 4 A; Table S1). Precipitates of a DBF-2-GFP fusion recovered all components of the central SIN cascade, including its regulatory subunit MOB-1, the two upstream kinases CDC-7 and SID-1 and its adaptor CDC-14, and the predicted GTPase SPG-1/NCU08878. In contrast, SID-1-GFP recovered only its subunit CDC-14 and the upstream components CDC-7 and SPG-1, but not the DBF-2/MOB-1 complex, indicating a less robust interaction with the SIN effector kinase. MST-1 did not co-purify these two kinases. However, CDC-7-GFP co-precipitated both GC kinases SID-1 and MST-1 in addition to the GTPase SPG-1 and the SID-1 adapter CDC-14, while only CDC-7 co-purified with MST-1. Next, we determined physical interactions of the SIN components in yeast two hybrid (Y2H) assays (Figure 4 B). DBF-2 and SID-1 directly interacted with their regulatory subunits MOB-1 and CDC-14, respectively, and both kinases were able to form homo-dimers. In addition, a weak interaction was detected between the two kinases. Interestingly, the upstream kinase CDC-7 did not directly interact with SID-1, but was connected to the GC kinase via its adaptor CDC-14. Finally, we detected a physical interaction of MST-1 and CDC-7 in Y2H tests (Figure 4 B), which was confirmed by co-immunoprecipitation (co-IP) experiments (Figure 4 C).

Our GFP-trap + MS data indicated that SID-1 and MST-1 only co-purified together, when we used CDC-7 as bait, while the two GC kinases behaved mutually exclusive in the other co-purifications. Moreover, we were unable to co-purify SPG-1, CDC-14 and the DBF-2/MOB-1 complex with MST-1, suggesting the presence of distinct CDC-7/SID-1 and CDC-7/MST-1 complexes. In order to further strengthen this assumption, we performed *in vitro* displacement assays, in which a GFP-trapped CDC-7-GFP/HA-GC kinase complex precipitated from a strain co-expressing both tagged kinases was incubated with excessive amounts of separately purified second GC kinase (Figure 4 D, E). The composition of the resulting CDC-7 complex was determined by Western blot analysis after pelleting the sepharose bead-associated proteins. In these assays, we were able to replace CDC-7-associated SID-1 with MST-1 and *vice versa*, indicating that the two GC kinases form mutually exclusive assemblies with CDC-7.

**Figure 5:**
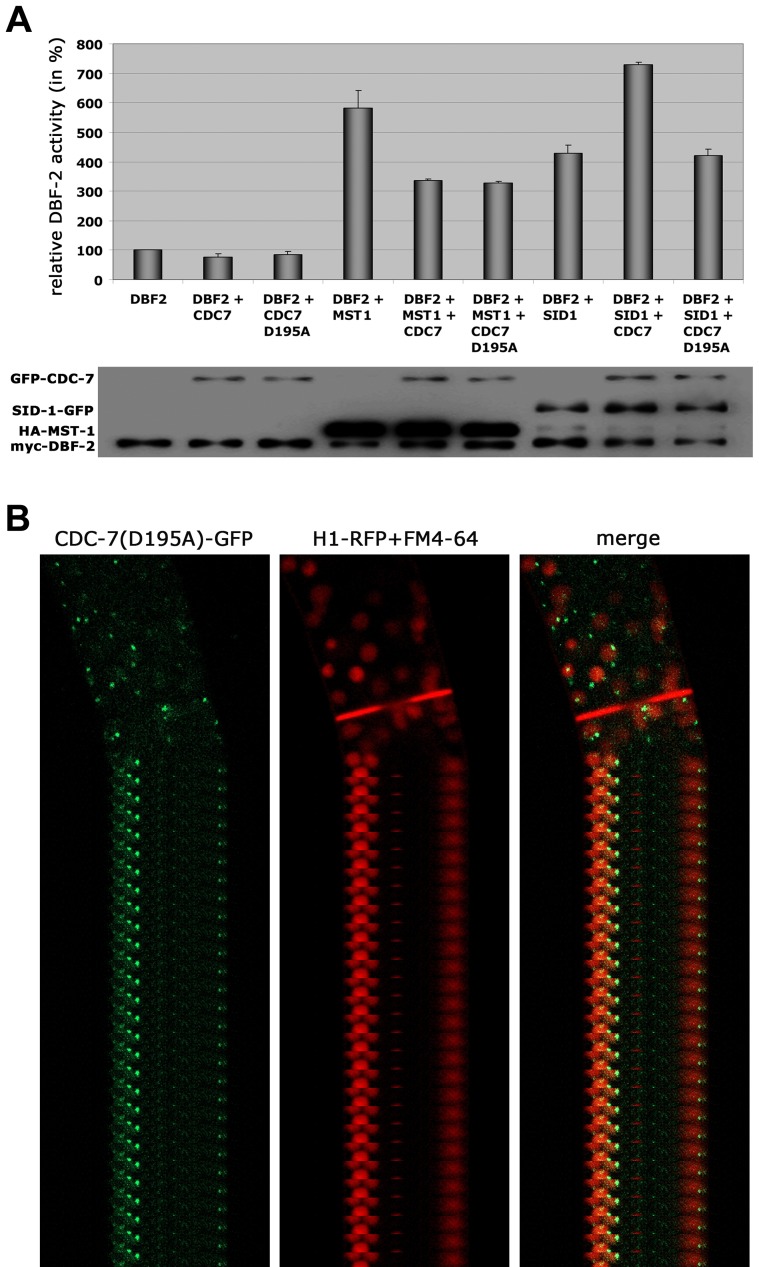
The regulation of MST-1 and SID-1 by CDC-7 is based on distinct mechanisms. (**A**) *In vitro* DBF-2 activity assays after addition of the indicated kinases. MST-1-stimulated DBF-2 activity is inhibited by addition of CDC-7 or CDC-7(D195A), while SID-1-stimulation requires active CDC-7. Western blot analysis of the precipitated proteins was used to determine comparable kinase levels (n = 4). (**B**) A GFP fusion construct of wt CDC-7 localized to spindle pole bodies and accumulated around the mature septal pore, while a CDC7(D195A)-GFP construct exclusively localized to SPBs. Nuclei were labeled with histone H1-RFP and plasma membrane with FM4-64.

Based on these interaction data, we hypothesized that the two GC kinases function in parallel as part of the SIN. Consistent with this prediction, we determined that purified SID-1 and MST-1 both stimulated DBF-2 activity *in vitro* (Figure 5 A). As predicted for the stepwise function of the SIN kinase cascade, addition of CDC-7 to the SID-1/DBF-2 kinase mixture further stimulated DBF-2, while addition of kinase-dead CDC-7(D195A) did not. In contrast, CDC-7 inhibited MST-1-driven stimulation of DBF-2, and this inhibition did not require functional CDC-7. As control, we confirmed that the kinase activity of CDC-7 was essential for complementation of the deletion defects of Δ*cdc-7* (data not shown). Strikingly, we found that septum association of CDC-7, but not its localization at SPBs was dependent on the functionality of CDC-7 (Figure 5 B). In contrast, the localization of DBF-2 and MST-1 at SPBs and septa did not require enzyme activity (Figure S4), potentially because homo-dimerization of the kinase-dead version with a functional kinase variant allowed correct localization in a wt background.

### MST-1 regulates the MOR network by two distinct mechanisms

**Figure 6:**
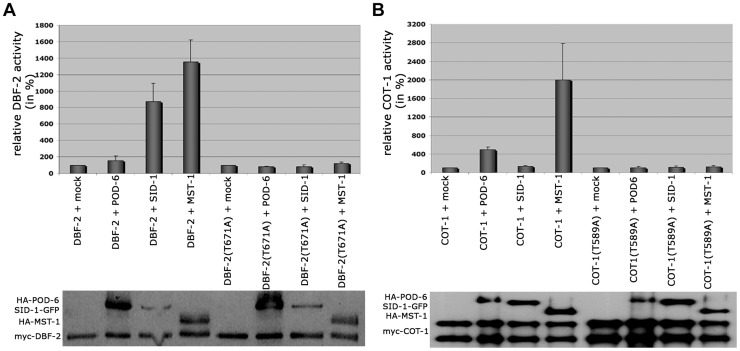
SID-1 and POD-6 are pathway-specific activators of the SIN and MOR, while MST-1 regulates both NDR kinase pathways. (**A**) *In vitro* kinase assays of precipitated MST-1 and SID-1 specifically stimulated DBF-2, but not DBF-2(T671A). (**B**) COT-1 was specifically phosphorylated by the upstream GC kinases POD-6 and MST-1, but not by SID-1. Western blot analysis of the precipitated proteins was used to determine comparable kinase levels.

Based on the presented genetic, biochemical and life imaging analyses, we conclude that MST-1 is part of the SIN and functions in parallel to SID-1. However, the hyperseptation defects observed in Δ*mst-1* partly phenocopied characteristics of *MOR* mutants. Consistent with the hypothesis that MST-1 may regulate both NDR kinase pathways was the ability of purified MST-1 to activate COT-1 in addition to its activation of DBF-2 (Figure 6 A, B). As control, we confirmed that SID-1 only stimulated DBF-2, while POD-6 was specific for COT-1, confirming that MST-1 has a specific function as promiscuous activator of both pathways. Moreover, MST-1 was unable to stimulate DBF-2 and COT-1 variants, in which their hydrophobic motif phosphorylation sites were modified, indicating that MST-1 activates both target kinases by hydrophobic motif phosphorylation.

**Figure 7:**
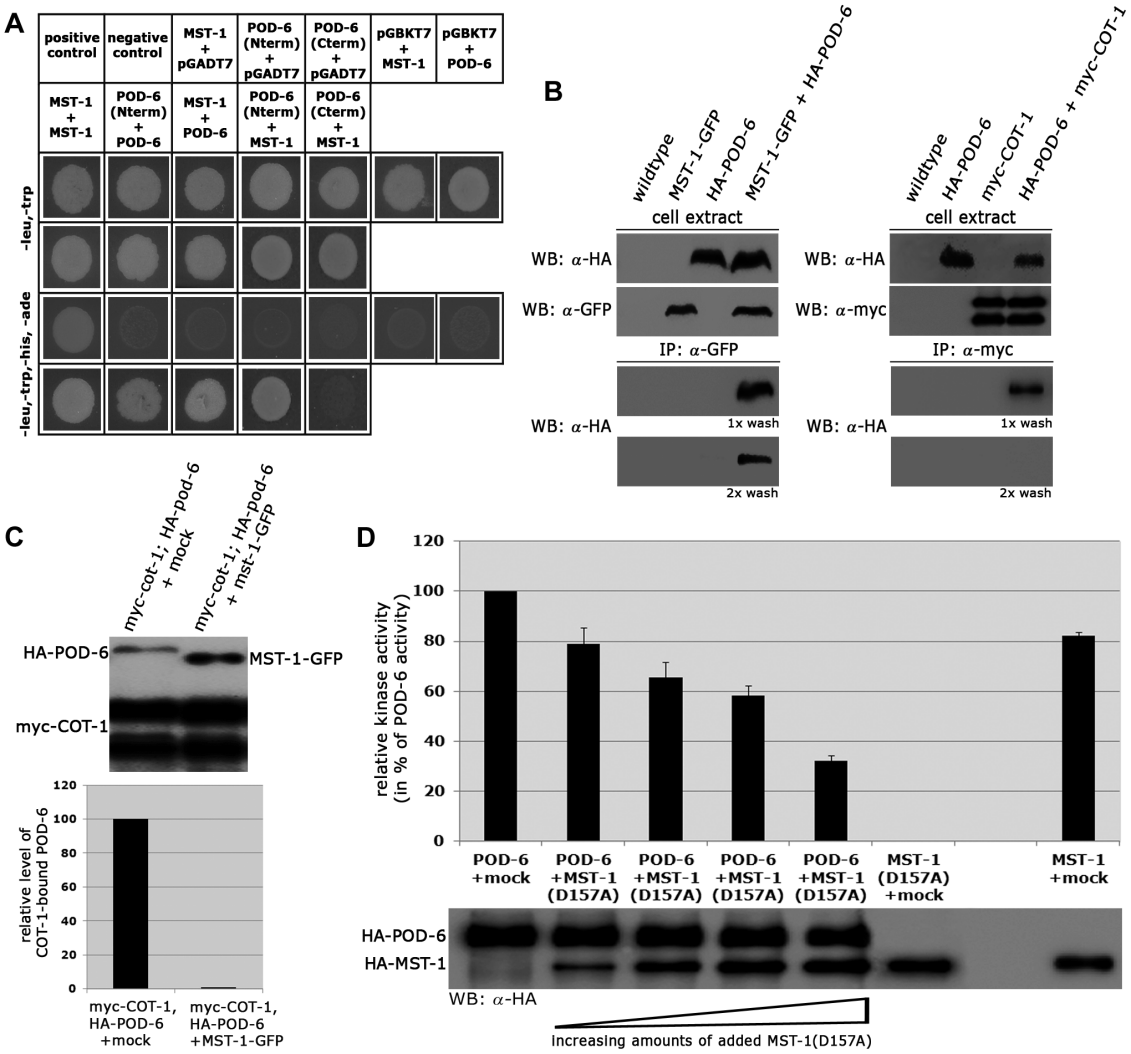
MST-1/POD-6 hetero-dimerization inhibits POD-6. (**A**) Yeast two-hybrid tests indicate hetero-dimerization of MST-1 and POD-6. (**B**) Co-immunoprecipitation experiments of MST-1-GFP and HA-POD-6 and of myc-COT-1 and HA-POD-6 from cell extracts co-expressing both functionally tagged proteins indicate interaction of the two kinase pairs. However, the MST-1/POD-6 interaction was more stable and withstood two washes with IP buffer, while the interaction between COT-1 and POD-6 was abolished under these conditions. (**C**) Protein displacement assay of a precipitated myc-COT-1/HA-POD-6 complex by the addition of separately purified MST-1 kinase, but not a mock-IP precipitate abolished the COT-1/POD-6 interaction. The upper panel displays a representative experiment, while three independent experiments are quantified in the lower graph. (**D**) POD-6 activity was determined *in vitro* in the presence of increasing levels of added kinase-dead MST-1(D157A) (n = 4).

Because we had detected several POD-6-specific peptides in one of the two MST-1-GFP GFP-trap+MS analyses (Table S1), we asked if MST-1 also interacted with the MOR pathway. Y2H experiments confirmed the physical interaction of MST-1 with POD-6 as well as the capability of both kinases to form homo-dimers through their kinase domains (Figure 7 A). Moreover, co-IP experiments suggested that the MST-1/POD-6 complex was more stable and withstood to more stringent wash conditions than the COT-1/POD-6 interaction (Figure 7 B). Moreover, POD-6 was replaced by MST-1 by adding separately purified MST-1 to the COT-1/POD-6 complex (Figure 7 C). Thus, a second mechanism of MST-1-dependent regulation of the MOR could involve dimerization of POD-6 and MST-1 and thereby inactivation of the GC kinase hetero-dimer. Indeed, when we titrated purified POD-6 with increasing levels of a kinase-dead version of MST-1(D157A) the activity of POD-6 decreased, while addition of a mock precipitate did not interfere with POD-6 activity (Figure 7 D).

## Discussion

**Figure 8:**
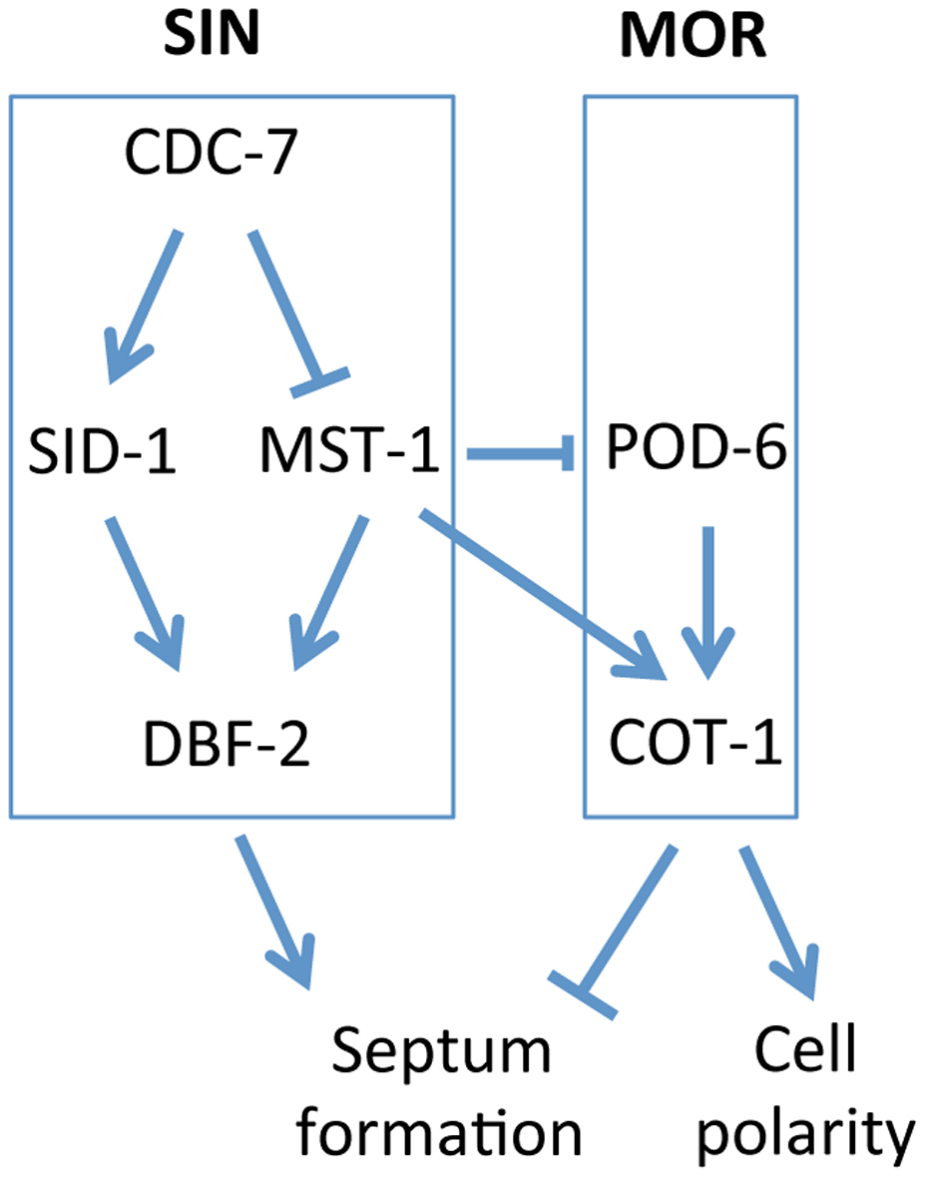
Model for the proposed interaction of the SIN and MOR networks during septation in *N. crassa*. See text for details.

GC kinases function as central components of the SIN and MOR networks, which have antagonistic functions in the regulation of septation in unicellular as well as filamentous fungi [5], [8], [20]. In this study, we report the characterization of MST-1, the third type III GC kinase present in fungi, and dissect its dual function during SIN and MOR signaling (summarized in Figure 8). Our results demonstrate that MST-1 is a SIN-associated kinase that physically interacts with CDC-7 and functions in parallel to SID-1 to activate DBF-2. However, the interaction data strongly suggest that MST-1 is not part of the central SIN cascade, but forms a distinct complex with CDC-7. As predicted for the tripartite kinase cascade, active CDC-7 is required to stimulate hydrophobic motif phosphorylation of DBF-2 by SID-1. In contrast, MST-1 activity is negatively regulated through CDC-7 in a mechanism that does not require active kinase, indicating that the regulation of SID-1 and MST-1 through CDC-7 is based on distinct mechanisms. A possible function of MST-1 in fine-tuning the SIN may be achieved in the generation of an incoherent type 4 feedforward loop through the two, parallel functioning, but negatively regulated CDC-7/SID-1 and CDC-7/MST-1 complexes that together activate DBF-2. This rarely found regulatory motif may allow for adaptive and thus robust SIN activity [54]. This hypothesis is supported by the phenotypic characteristics of *N. crassa SIN* mutants, which revealed less severe defects of *sid-1* mutants compared to strains deficient for *cdc-7* or *dbf-2* [43]. In summary, these results indicate that the composition and function of the SIN as a linear kinase cascade represents a simplified view. Our biochemical data also imply that the CDC-7/MST-1 complex may function independently of the GTPase SPG-1, a speculation that requires further exploration.

The maturation of the CAR in *N. crassa* is driven by the coalescence of a filamentous actin meshwork, called the SAT [50], [52], [53]. The association of all SIN components except CDC-7 approximately 3 min prior to the start of CAR constriction coincides with the time window of SAT to CAR transition, and thus the SIN is likely involved in this process. However, the aseptate nature of *sin* mutants precludes a detailed analysis of CAR function in these strains. Here we show that the SIN-associated kinase MST-1 plays a significant role in the proper assembly and constriction of the CAR. The defects generated by *mst-1* mutants allow visualizing defects during the transition of SAT to CAR and imply a central function of the SIN in CAR maturation and in CAR constriction. Life imaging of actin as well as formin dynamics revealed two striking defects in Δ*mst-1*. First, the CAR was frequently generated in an asymmetric manner, resulting in its acentric constriction and the formation of mispositioned septal pores. Moreover, the transition from the SAT to the CAR at the future septation site was delayed about 3-4-fold compared to wt cells, and the SAT frequently failed to coalesce into a functional CAR, generating constriction-defective spirals. In summary, we speculate that this complex phenotype is the result of mis-regulated septum initiation; i.e. the trigger required for septum placement/initiation may not be terminated, but can signal into the neighboring area to allow the sequential formation of septa and/or spirals if the SAT does not properly coalesce into a functional CAR (Video S4).

All SIN kinases, including MST-1, also localize in a cell cycle-independent manner to both SPBs. This pattern differs from the localization of homologous kinases in budding and fission yeast. In these unicellular models, only the terminal kinase constitutively associates with both SPBs and relocates after its activation at the SPB to the septum, while the upstream kinases associate only with one SPB in a cell cycle-dependent manner and do not relocate [10], [55]. This re-localization of the activated Ndr kinase is thought to be important for controlling the coordination between cell cycle progression and cell division. Strikingly, SIN activation in *A. nidulans* does not require its previous interaction with the SPB [32], and localization of the entire SIN at SPBs is cell cycle independent in *N. crassa*. Moreover, we found that the activity of CDC-7 is essential for its localization to septa, but not its association with the SPB. These data strongly suggest differential regulation of the SIN in uninuclear and syncytial fungi. The mechanism for positioning new septa in a multi-nuclear syncytium is currently unresolved [27], [28]. Although the SIN is a prime candidate for transmitting nuclear signals that are required for the selection of future septation sites [27], [28], [30], we have currently no information if SPB association of the SIN has any function during vegetative hyphal growth.

MST-1 also interacts with and regulates several components of the MOR network. First, MST-1 activates the MOR effector kinase COT1 through hydrophobic motif phosphorylation, a mechanism previously shown for other fungal and animal NDR kinases [41], [43], [56]–[58]. This dual function of SIN and MOR regulation reflects NDR kinase signaling in higher eukaryotes, where the GC kinase Hippo has been shown to activate two NDR kinases that regulate cell proliferation as well as polar morphogenesis, respectively [59]–[61]. Moreover, MST-1 can form heterodimers with the MOR kinase POD-6, and this interaction results in inhibition of the GC kinase complex. Thus, MST-1 can stimulate as well as inhibit MOR signaling. Although the functional significance of this dual function of MST-1 is yet unresolved, the *N. crassa* MOR likely has multiple functions during septation. First, *MOR* mutants are characterized by abundant, closely spaced septa, arguing for an early function of the MOR in the inhibition of septum initiation. However, COT1, its regulatory subunit MOB-2A, and POD-6 associate with the CAR only after the start of septum constriction and primarily label the mature septal pore (this study; [41], [42]). Thus, the MOR may also be critical to regulate termination of constriction and proper pore formation/maintenance. Thus, although originally thought to constitute distinct modules, antagonistic cross-talk between the SIN and MOR occurs at multiple levels (this study; [19], [21]). Further analysis is required to dissect their individual impact for the coordinated action of both networks during cell division.

## Materials and Methods

### Strains, media and growth conditions

Strains used in this study are listed in Table S2. Genetic procedures and media used for handling *N. crassa* are available through the Fungal Genetic Stock Center (www.fgsc.net; [62]).

### Two hybrid plasmids and methods

The Matchmaker Two-Hybrid system 3 (Clontech, USA) was used according to the manufacturer's instructions. cDNA of genes of interest for two hybrid tests was amplified with primers listed in Table S3 spanning the coding region from start to stop codon as annotated by the *N. crassa* database (http://www.broadinstitute.org/annotation/genome/neurospora/MultiHome.html) and cloned either into the pGADT7 vector containing the GAL4 activation domain or into pGBKT7 containing the DNA-binding domain. Because the pGBKT7-POD-6 fusion protein was determined to be self-activating, we generated two fragments containing the N-terminal kinase domain (aa 1–412) and C-terminal non-catalytic domain of POD-6 (aa 415–929). Fusion proteins were (co-) expressed in *S. cerevisiae* AH109 and potential interactions determined by growth tests on SD medium lacking the amino acids adenine, histidine, leucine and tryptophan or on SD medium supplemented with 10 mM 3-amino-1,2,4-triazole and lacking leucine, tryptophane and histidine.

### Plasmid construction and fungal expression of tagged proteins

To obtain strains for the subcellular localization and expression of GFP-tagged POD-6 and HA/GFP-tagged MST-1 fusion proteins from the *his-3* locus, their open reading frames were amplified by PCR as annotated by the *N. crassa* database using primers listed in Table S3 and introduced via *SpeI/PacI*, *BamHI/EcoRI* or *XbaI/BamHI*, respectively, into pMF272 or pHAN1 [63]–[65]. The kinase dead versions of CDC-7 and MST-1 were generated by site-directed mutagenesis according to the manufacturer's instructions (Stratagene). The final plasmids were transformed into *his-3*, *nic-3*;*his-3* or *trp-1*;*his-3* and were selected for complementation of the *his-3* auxotrophy. Strains expressing the fusion constructs were crossed with the respective deletion strain and the progeny assayed for hygromycin resistance, GFP expression and complementation of the mutant growth/septation defects. Alternatively, histidine auxotrophic deletion strains were generated and directly transformed with plasmids allowing expression of the fusion proteins from the *his-3* locus, and under control of the inducible P*ccg-1* promoter.

### Microscopy

Low magnification analysis of fungal hyphae or colonies was performed as described [66], [67] using an SZX16 stereomicroscope, equipped with a Colorview III camera and Cell^D^ imaging software (Olympus). In order to avoid accumulation of suppressor mutations, aseptate deletion strains were maintained as heterokaryons. An inverted Axiovert Observer Z1 microscope (Zeiss) equipped with a CSU-X1 A1 confocal scanner unit and a QuantEM 512SC camera (Photometrics) was used for spinning disk confocal microscopy [68]. Slidebook 5.0 software (Intelligent Imaging Innovations) was used for image/video acquisition, deconvolution and image analysis. Cell wall and plasma membrane were stained with Calcofluor White (2 µg/ml-1) and FM4-64 (1 µg/ml-1) respectively. Time-lapse imaging was performed at capture intervals of 20–30 s for periods up to 15 min using the oil immersion objective 100×/1.3. Image series were converted into movies (*.movs). For confocal live-cell imaging, we used an inverted LSM-510 Meta laser scanning microscope (Zeiss) equipped with an argon ion laser for excitation at 488 nm wavelength and GFP filters for emission at 515–530 nm and a 63× (PH3)/1.4 N.A. oil immersion objective [69]. Laser intensity was kept to a minimum (1.5%) to reduce photobleaching and phototoxic effects. Time-lapse imaging was performed at scan intervals of 3 to 15 s for periods up to 40 min. Image resolution was 512×512 pixels and 300 dpi. Confocal images were captured using LSM-510 software and evaluated with an LSM 510 Image Examiner.

### Protein methods

Liquid *N. crassa* cultures were grown at room temperature, harvested gently by filtration using a Büchner funnel and ground in liquid nitrogen. The pulverized mycelium was mixed 1∶1 with IP-Buffer (50 mM Tris/HCL pH 7.5, 100 mM KCl, 10 mM MgCl_2_, 0.1% NP-40, 5 mM NaF, 1 mM PEFAbloc SC, 2 mM DTT, 1 mM Na_3_VO_4_, 25 mM β-glycerophosphate, 2 mM benzamidine, 2 ng/µl pepstatin A, 10 ng/µl aprotinin, 10 ng/µl leupeptin) and centrifuged twice at 4°C (15 min at 4500 g and 30 min at 14000 g) to obtain crude cell extracts. Co-immunoprecipitation experiments were performed with cell extracts from fused, heterokaryotic strains that were selected by their ability to grow on minimal media lacking supplements. Protein-decorated beads were washed twice with IP buffer, and immunoprecipitated proteins were recovered by boiling the beads for 10 min at 98°C in 3× Laemmli buffer and separated by 10% SDS-PAGE. Monoclonal mouse α-HA (clone HA-7, Sigma Aldrich), α-myc (9E10, Santa Cruz) and α-GFP (B2, Santa Cruz) were used in this study. Protein displacement from CDC-7-containing complexes was assayed by precipitating CDC-7-GFP from cell extracts co-expressing HA-SID-1 or HA-MST-1, respectively, and mixed with separately purified MST-1-GFP or SID-1-GFP, respectively. Precipitates of a mock-IP from wt extracts were used as controls. Samples were incubated for 30 min at RT and the suspensions subsequently centrifuged (2 min at 4000 g) to remove supernatant and washed once with IP buffer to remove displaced/unbound HA-SID-1. Immunoprecipitated proteins were recovered by boiling sepharose beads for 10 min at 98°C in 3× Laemmli buffer. GFP-trap experiments, mass spectrometry and database analysis were performed as described [68], [70]. The GFP-trap pulldown reagent is a quality GFP-binding protein derived from alpaca coupled to a monovalent matrix and was obtained from Chromotek. COT-1 and DBF-2 kinase activity assays were performed as described [43], [58] according to a modified protocol described previously for animal NDR kinase [71]. NDR kinase stimulation by upstream kinases was assayed as described [43]. GC kinase activity was determined by phosphorylation of the peptide substrate EESPELSLPFIGYTFKRFDNNFR, which was used at a final concentration of 2 mM [41]. Assay conditions were analogous to NDR kinase assays described above.

## Supporting Information

Figure S1 Phylogram of fungal GC kinases. The tree was generated by using ClustalX 2.1 with bootstrap support (111 random number generator seed and 1000 bootstrap trials) and the predicted protein sequences from selected ascomycete (*Saccharomyces cerevisiae*, *Candida albicans*, *Ashbya gossypii*, *Yarrowia lipolytica*, *Schizosaccharomyces pombe*, *Schizosaccharomyces japonicus*, *Neurospora crassa*, *Aspergillus nidulans*, *Magnaporthe grisea*, *Fusarium graminearum*, *Botrytis cinerea*, *Histoplasma capsulatum*), basidiomycete *(Phanerochaetae chrysosporium*, *Coprinus cinereus*, *Ustilago maydis*, *Cryptococcus neoformans)* and zygomycete (*Rhizopus oryzae*) proteins. *S. cerevisiae* Cdc15 was used as outgroup member (multiple alignment parameters: gap opening 50.0, gap extension 50.0, delay divergent sequences 30%, protein weight matrix gonnet series). The red, blue and yellow boxes label GC kinases with homologies to *N. crassa* POD-6, MST-1 and SID-1, respectively. The predicted *R. oryzae* protein HMPREF1544_07988 likely represents an aberrant SID-1 homolog.
(PDF)

Figure S2 Localization of POD-6 at the hyphal tip. A GFP fusion construct of POD-6 localized at the hyphal tip in a dot-like structure in the distal region of the *Spitzenkörper* and as membrane-associated apical crescent. Plasma membrane and *Spitzenkörper* were labeled with FM4-64.
(PDF)

Figure S3 Δ*mst-1* displays synthetic interactions with *SIN*, but not *MOR* pathway mutants. (**A**) Δ*mst-1*×wt crosses produced a large number of round ascospores, in contrast to the typical pea-shaped ascospores generated in wt×wt crosses. Δ*mst-1*×Δ*mst-1* crosses were blocked after perithecium formation, resulting in fruiting bodies that lacked most asci and all ascopores. (**B**) Synthetic defects were observed in crosses of Δ*mst-1* with *SIN* but not *MOR* mutants. Δ*mst-1*×Δ*dbf-2* and Δ*mst-1*×Δ*sid-1* crosses generated empty perithecia. In contrast, Δ*mst-1*×Δ*cot-1* and Δ*mst-1*×Δ*pod-6* crosses resulted in the expected segregation of round and normally shaped ascospores.
(PDF)

Figure S4 Localization of the inactive kinase variants DBF-2(D422A) and MST-1(D157A) at spindle pole bodies and constricting septa. Nuclei and plasma membrane are co-labeled with histone H1-RFP and FM4-64, respectively.
(PDF)

Table S1 Identification of *N. crassa* SIN proteins by mass spectrometry.
(XLSX)

Table S2 *N. crassa* strains used in this study.
(DOCX)

Table S3 Primers used in this study.
(DOCX)

Video S1 Time-course of MST-1-GFP localization during septum formation. MST-1-GFP formed cortical rings at incipient septation sites that constricted during septum formation and accumulated around the septal pore of the completed septum (**a**) GFP channel; (**b**) RFP channel; (**c**) merged. The plasma membrane was stained with FM4-64. Images were captured at 20 sec intervals.
(MOV)

Video S2 4D reconstruction of z-stacks in time lapse series of the BNI-1-GFP-labeled CAR in the wt. BNI-1-GFP developed concentrically constricting rings, resulting in centrally positioned septal pores. The cell wall was stained with Calcofluor White. Images were captured at 120 s intervals.
(MOV)

Video S3 4D reconstruction of z-stacks in time lapse series of the BNI-1-GFP-labeled CAR in Δ*mst-1*. BNI-1 formed asymmetric, open rings that led to acentric constriction and asymmetric septal pores. The cell wall was stained with Calcofluor White. Images were captured at 90 s intervals.
(MOV)

Video S4 Time-course of BNI-1-GFP localization during septum formation in Δ*mst-1*. During the formation of multiple, closely spaced septa, BNI-1-GFP formed cortical, centrically and acentrically constricting rings and accumulated around the septal pore of the completed septum. Multiple, closely spaced septa can be formed in a simultaneous as well as sequential manner. The plasma membrane was stained with FM4-64. Images were captured at 20 sec intervals.
(MOV)

Video S5 4D reconstruction of z-stacks in time lapse series of the Lifeact-GFP-labeled CAR in wt cells. Lifeact-GFP labeled a mesh of F-actin cables and patches around the future septation site, which subsequently coalesced to form the CAR. Images were captured at scan intervals of 0.5 to 3 s for periods up to 40 min.
(MOV)

Video S6 4D reconstruction of z-stacks in time lapse series of the Lifeact-GFP-labeled CAR in Δ*mst-1*. Failure of correct CAR assembly resulted in acentric constriction and asymmetric position of the septal pore. Images were captured at scan intervals of 0.5 to 3 s for periods up to 40 min.
(MOV)

Video S7 4D reconstruction of z-stacks in time lapse series of the Lifeact-GFP-labeled CAR in Δ*mst-1*. The F-actin meshwork was mis-organized and the actin cables were irregularly distributed in Δ*mst-1*. Failure of correct CAR assembly resulted in the formation of open actin spirals, which were unable to constrict. Images were captured at scan intervals of 0.5 to 3 s for periods up to 40 min.
(MOV)
