# Supplementary material for: Proper Actin Ring Formation and Septum Constriction Requires Coordinated Regulation of SIN and MOR Pathways through the Germinal Centre Kinase MST-1
Source: PLoS Genet. 2014 Apr 24;10(4):e1004306. doi: 10.1371/journal.pgen.1004306 (PMC3998894; doi:10.1371/journal.pgen.1004306)
Supplement: Figure S1 — Phylogram of fungal GC kinases. The tree was generated by using ClustalX 2.1 with bootstrap support (111 random number generator seed and 1000 bootstrap trials) and the predicted protein sequences from selected ascomycete (Saccharomyces cerevisiae, Candida albicans, Ashbya gossypii, Yarrowia lipolytica, Schizosaccharomyces pombe, Schizosaccharomyces japonicus, Neurospora crassa, Aspergillus nidulans, Magnaporthe grisea, Fusarium graminearum, Botrytis cinerea, Histoplasma capsulatum), basidiomycete (Phanerochaetae chrysosporium, Coprinus cinereus, Ustilago maydis, Cryptococcus neoformans) and zygomycete (Rhizopus oryzae) proteins. S. cerevisiae Cdc15 was used as outgroup member (multiple alignment parameters: gap opening 50.0, gap extension 50.0, delay divergent sequences 30%, protein weight matrix gonnet series). The red, blue and yellow boxes label GC kinases with homologies to N. crassa POD-6, MST-1 and SID-1, respectively. The predicted R. oryzae protein HMPREF1544_07988 likely represents an aberrant SID-1 homolog. (PDF) [file pgen.1004306.s001.pdf]

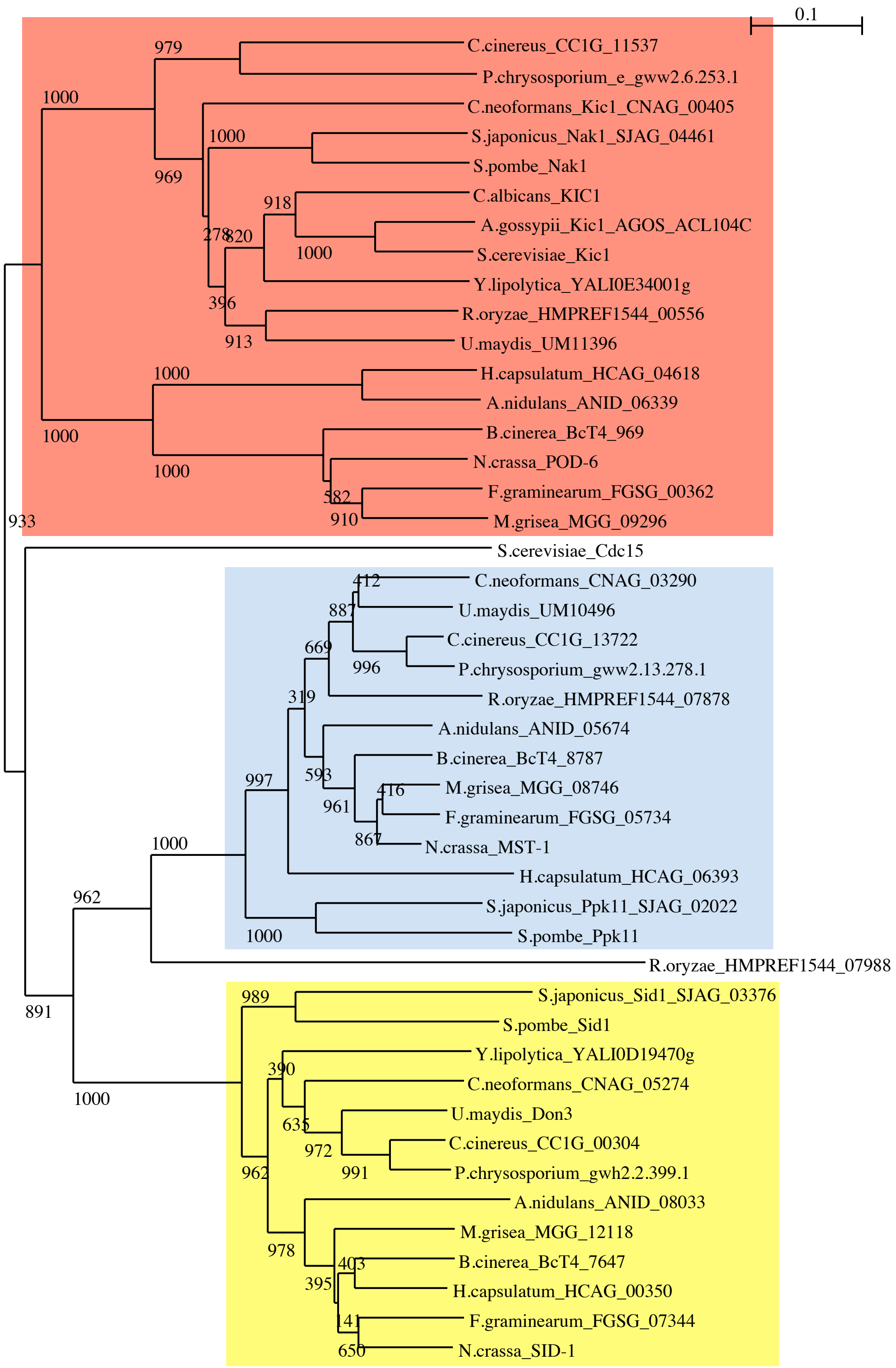

Figure S1. Phylogram of fungal GC kinases. The tree was generated by using ClustalX 2.1 with bootstrap support (111 random number generator seed and 1000 bootstrap trials) and the predicted protein sequences from selected ascomycete (*Saccharomyces cerevisiae, Candida albicans, Ashbya gossypii, Yarrowia lipolytica, Schizosaccharomyces pombe, Schizosaccharomyces japonicus, Neurospora crassa, Aspergillus nidulans, Magnaporthe grisea, Fusarium graminearum, Botrytis cinerea, Histoplasma capsulatum),* basidiomycete *(Phanerochaetae chrysosporium, Coprinus cinereus, Ustilago maydis, Cryptococcus neoformans)* and zygomycete (*Rhizopus oryzae*) proteins. *S. cerevisiae* Cdc15 was used as outgroup member (multiple alignment parameters: gap opening 50.0, gap extension 50.0, delay divergent sequences 30%, protein weight matrix gonnet series). The red, blue and yellow boxes label GC kinases with homologies to *N. crassa* POD-6, MST-1 and SID-1, respectively. The predicted *R. oryzae* protein HMPREF1544_07988 likely represents an aberrant SID-1 homolog.
