# Supplementary material for: Proper Actin Ring Formation and Septum Constriction Requires Coordinated Regulation of SIN and MOR Pathways through the Germinal Centre Kinase MST-1
Source: PLoS Genet. 2014 Apr 24;10(4):e1004306. doi: 10.1371/journal.pgen.1004306 (PMC3998894; doi:10.1371/journal.pgen.1004306)
Supplement: Figure S2 — Localization of POD-6 at the hyphal tip. A GFP fusion construct of POD-6 localized at the hyphal tip in a dot-like structure in the distal region of the Spitzenkörper and as membrane-associated apical crescent. Plasma membrane and Spitzenkörper were labeled with FM4-64. (PDF) [file pgen.1004306.s002.pdf]

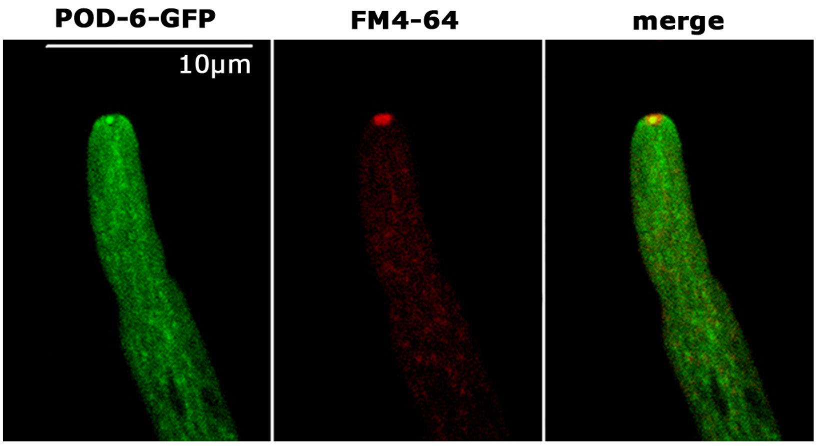

Figure S2. Localization of POD-6 at the hyphal tip. A GFP fusion construct of POD-6 localized at the hyphal tip in a dot-like structure in the distal region of the *Spitzenkörper* and as membrane-associated apical crescent.
