# Supplementary material for: Proper Actin Ring Formation and Septum Constriction Requires Coordinated Regulation of SIN and MOR Pathways through the Germinal Centre Kinase MST-1
Source: PLoS Genet. 2014 Apr 24;10(4):e1004306. doi: 10.1371/journal.pgen.1004306 (PMC3998894; doi:10.1371/journal.pgen.1004306)
Supplement: Figure S3 — Δmst-1 displays synthetic interactions with SIN, but not MOR pathway mutants. (A) Δmst-1×wt crosses produced a large number of round ascospores, in contrast to the typical pea-shaped ascospores generated in wt×wt crosses. Δmst-1×Δmst-1 crosses were blocked after perithecium formation, resulting in fruiting bodies that lacked most asci and all ascopores. (B) Synthetic defects were observed in crosses of Δmst-1 with SIN but not MOR mutants. Δmst-1×Δdbf-2 and Δmst-1×Δsid-1 crosses generated empty perithecia. In contrast, Δmst-1×Δcot-1 and Δmst-1×Δpod-6 crosses resulted in the expected segregation of round and normally shaped ascospores. (PDF) [file pgen.1004306.s003.pdf]

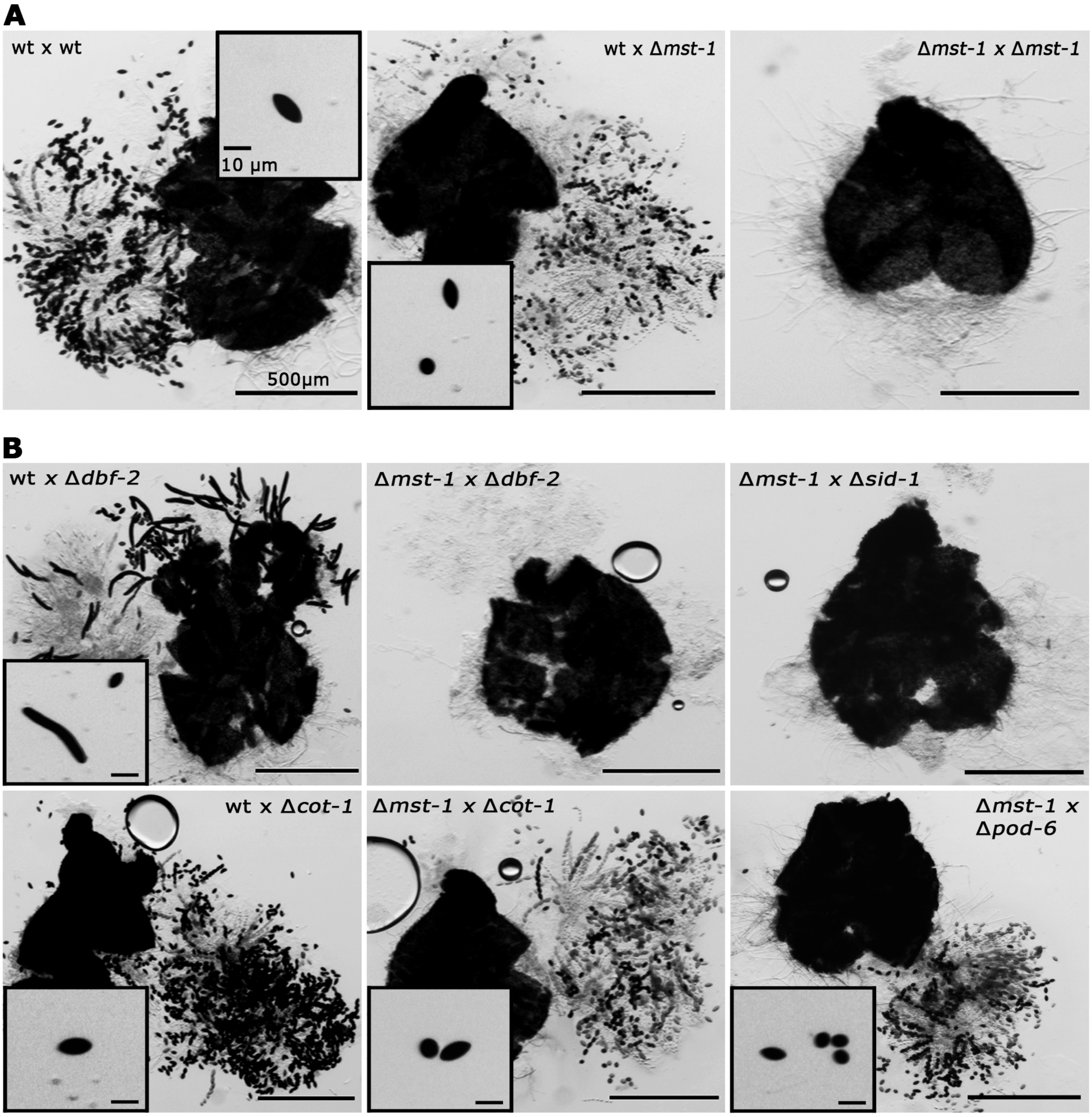

Figure S3. Δmst-1 displays synthetic interactions with SIN, but not MOR pathway mutants. **(A)** Δ*mst-1* x wild type crosses produced a large number of round ascospores, in contrast to the typical pea-shaped ascospores generated in wild type x wild type crosses. Δ*mst-1* x Δ*mst-1* crosses were blocked after perithecium formation, resulting in fruiting bodies that lacked most asci and all ascopores. **(B)** Synthetic defects were observed in crosses of Δ*mst-1* with *SIN* but not *MOR* mutants. Δ*mst-1* x Δ*dbf-2* and Δ*mst-1* x Δ*sid-1* crosses generated empty perithecia. In contrast, Δ*mst-1* x Δ*cot-1* and Δ*mst-1* x Δ*pod-6* crosses resulted in the expected segregation of round and normally shaped ascospores.
