# Supplementary material for: Proper Actin Ring Formation and Septum Constriction Requires Coordinated Regulation of SIN and MOR Pathways through the Germinal Centre Kinase MST-1
Source: PLoS Genet. 2014 Apr 24;10(4):e1004306. doi: 10.1371/journal.pgen.1004306 (PMC3998894; doi:10.1371/journal.pgen.1004306)
Supplement: Figure S4 — Localization of the inactive kinase variants DBF-2(D422A) and MST-1(D157A) at spindle pole bodies and constricting septa. Nuclei and plasma membrane are co-labeled with histone H1-RFP and FM4-64, respectively. (PDF) [file pgen.1004306.s004.pdf]

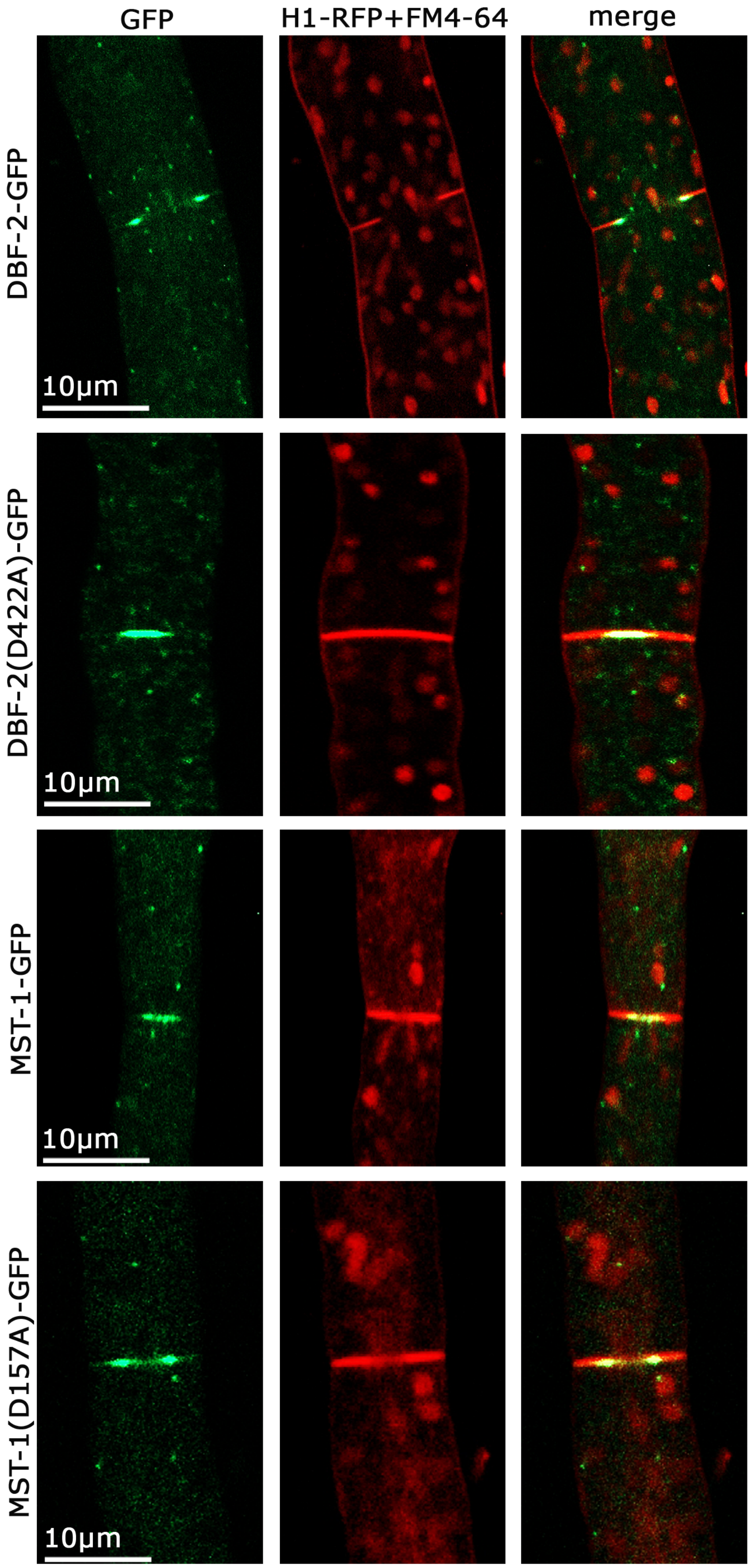

Figure S4. Localization of the inactive kinase variants DBF-2(D422A) and MST-1(D157A) at spindle pole bodies and constricting septa. Nuclei and plasma membrane are co-labeled with H1-RFP and FM4-64, respectively.
