# Supplementary material for: Proper Actin Ring Formation and Septum Constriction Requires Coordinated Regulation of SIN and MOR Pathways through the Germinal Centre Kinase MST-1
Source: PLoS Genet. 2014 Apr 24;10(4):e1004306. doi: 10.1371/journal.pgen.1004306 (PMC3998894; doi:10.1371/journal.pgen.1004306)
Supplement: Table S2 — N. crassa strains used in this study. (DOCX) [file pgen.1004306.s006.docx]

**Table S2: *N. crassa* strains used in this study**

| *Strain* | *Genotype* | Source |
| --- | --- | --- |
| *wild type 74* | *OR231 Mat A* | FGSC #987 |
| *wild type ORS* | *SL6 Mat a* | FGSC #4200 |
| *his-3 A* | *his-3 Mat A* | FGSC #6103 |
| *his-3 a* | *his-3 Mat a* | FGSC #718 |
| *trp-1;his-3* | *trp-1- his-3-* | [[30](#_ENREF_30)] |
| *nic-3;his-3* | *nic-3- his-3-* | [[30](#_ENREF_30)] |
| *∆dbf-2* | *hph::dbf-2∆* | [[30](#_ENREF_30)] |
| *∆cdc-7* | *hph::cdc-7∆* | [[34](#_ENREF_34)] |
| *∆sid-1* | *hph::sid-1∆* | [[34](#_ENREF_34)] |
| *∆mst-1* | *hph::mst-1∆* | FGSC #11478 |
| *∆mst-1 his-3* | *hph::mst-1∆ his-3* | This study |
| *∆cot-1* | Δ*cot-1::hph^R^ + cot-1^+^* Δ*mus51::bar^R^ a* | FGSC #14525 |
| *∆pod-6* | Δ*pod-6::natR* | [[29](#_ENREF_29)] |
| *pod-6(ts)* | *pod-6(I310K)* | [[27](#_ENREF_27)] |
| *gfp-dbf-2* | *Pccg-1-sgfp-dbf-2::his-3 hph::dbf-2∆* | [[34](#_ENREF_34)] |
| *gfp-dbf-2(D422A)* | *Pccg-1-sgfp-dbf-2(D422A)::his-3* | [[34](#_ENREF_34)] |
| *cot-1-gfp* | *Pcot-1-cot-1-sgfp::hph* | [[32](#_ENREF_32)] |
| *pod-6-gfp* | *Pccg-1-pod-6-sgfp::his-3 hph::pod-6∆* | This study |
| *mst-1-gfp* | *Pccg-1-mst-1-sgfp::his-3 hph::mst-1∆* | This study |
| *mst-1-gfp(D157A)* | *Pccg-1-mst-1-sgfp(D157A)::his-3 hph::mst-1∆* | This study |
| *sid-1-gfp* | *Pccg-1-sid-1-sgfp::his-3 hph::sid-1∆* | [[34](#_ENREF_34)] |
| *cdc-7-gfp* | *Pccg-1-cdc-7-sgfp::his-3 hph::cdc-7∆* | [[34](#_ENREF_34)] |
| *cdc-7-gfp(D195A)* | *Pccg-1-cdc-7-sgfp(D195A)::his-3* | This study |
| *bni-1-gfp* | *Pccg-1-bni1-sgfp::his-3 hph::bni-1∆* | [[26](#_ENREF_26)] |
| *lifeact-gfp* | *Pccg-1-lifeact-egfp::his-3* | [[43](#_ENREF_43)] |
| *h1-rfp* | *Pccg-1-rfp-h1::his-3 mat* A | M. Freitag, USA |
| *myc-dbf-2* | *Pccg-1-myc-DBF-2::his-3 hph::dbf-2∆* | [[34](#_ENREF_34)] |
| *myc-dbf-2(T671A)* | *Pccg-1-myc-DBF-2(T671A)::his-3 hph::dbf-2∆* | This study |
| *myc-cot-1* | *Pcot-1-myc-cot-1* | [[49](#_ENREF_49)] |
| *myc-cot-1(T589A)* | *Pcot-1-myc-cot-1(T589)* | [[49](#_ENREF_49)] |
| *HA-pod-6* | Ppod-6-HA-pod-6; his-3 | [[32](#_ENREF_32)] |
| *HA-sid-1* | *Pccg-1-HA-sid-1::his-3 hph::sid-1∆* | This study |
| *HA-mst-1* | *Pccg-1-HA-mst-1::his-3 hph::mst-1∆* | This study |
| *HA-mst-1(D157A)* | *Pccg-1-HA-mst-1(D157A)::his-3* | This study |
| *mst-1-gfp;HA-pod-6* | *Pccg-1-HA-mst-1::*his-3 Ppod-6-HA-pod-6 | This study |
| *HA-pod-6;myc-cot-1;his-3* | *Pcot-1-myc-cot-1 Ppod-6-HA-pod6 his-3-* | [[33](#_ENREF_33)] |
| *bni-1-gfp;∆mst-1* | *Pccg-1-bni-1-sgfp:: hph::mst-1∆* | This study |
| *lifeact-gfp; ∆mst-1* | *Pccg-1-lifeact-egfp:: hph::mst-1∆* | This study |
| *HA-mst-1;trp-1* | *Pccg-1-HA-mst-1::his-3 trp-1-* | This study |
| *HA-sid-1;trp-1* | *Pccg-1-HA-sid-1::his-3 trp-1-* | [[34](#_ENREF_34)] |
| *cdc-7-gfp;nic-1* | *Pccg-1-cdc-7-gfp::his-3 nic-1-* | [[34](#_ENREF_34)] |
