# Supplementary material for: Proper Actin Ring Formation and Septum Constriction Requires Coordinated Regulation of SIN and MOR Pathways through the Germinal Centre Kinase MST-1
Source: PLoS Genet. 2014 Apr 24;10(4):e1004306. doi: 10.1371/journal.pgen.1004306 (PMC3998894; doi:10.1371/journal.pgen.1004306)
Supplement: Table S3 — Primers used in this study. (DOCX) [file pgen.1004306.s007.docx]

**Table S3. Primers used in this study**

| **Name** | **Sequence 5‘- 3‘** |
| --- | --- |
| point mutation constructs: |  |
| YH-CDC7-D2A-forw | ATG GCA CCG TCA AGT TAG CA*g cc*T TTG GCG TGT CAA CCA GCA C |
| YH-CDC7-D2A-rev | GTG CTG GTT GAC ACG CCA AA*g gc*T GCT AAC TTG ACG GTG CCA T |
| YH-Mst1-D2A-forw | CAG GTC AAG CTA GCT *gct* TTT GGT GTT TCC GG |
| YH-Mst1-D2A-rev | CCG GAA ACA CCA AA*a gc*A GCT AGC TTG ACC TG |
| 3xHA-tag constructs: |  |
| AD0772_BamHI5 | **gga tcc** ATG GCC GAC CGC G |
| AD0772_EcoRI3 | **gaa ttc** CTA ACC ACC ATG CCC |
| GFP-fusion plasmids: |  |
| AD0772_GFP_XbaI5 | **tct aga** GGA TGG CCG ACC GC |
| AD0772_GFP_BamHI3 | **gga tcc** CAC CAC CAT GCC C |
| YH-POD-6-ATG-Spe | **act agt** ATG GCG ACC CTA TCG |
| YH-POD-6-Stop-Pac | **tta att aa**G ACA CTC GTG TCC AC |
| yeast two hybrid constructs: |  |
| POD6 Nde-5 | GAT CAG **cat atg** GCG ACC CTA TCG GTA TAC |
| POD6 Eco-3 | G**ga att c**CT ACC TCC CTC AGA CAC TCG TG |
| AD6636_NdeI5 | **cat atg** GAG TCC CTA CTA TCG |
| AD6636_EcoRI3 | **gaa ttc** TCA GCT CAA CAC ACC |
| AD1335_NdeI5 | **cat atg** ATG GCG CCG AAC C |
| AD1335_NdeI3 | **cat atg** CTA CGA CCA CCT CAT GTC C |
| AD4096_EcoRI5 | **gaa ttc** ATG GCC GAC GAA GG |
| AD4096_EcoRI3 | **gaa ttc** CTA AGA TCC CGC AAC G |
| AD0772_NdeI5 | **cat atg** TGG GAC TTT GGC ACA G |
| AD0772_EcoRI3 | **gaa ttc** CTA GTC CTC ATT GAT ATG |
| SM9071 BamHI | GCC**ggatcc**CTACAGCATCGTACCAAAATTG |
| SM9071 EcoRI | GCC**gaattc**ATGTCTAGCTACTTGACAAACTTC |
| SM1605 EcoRI | G**gaattc**ATGAGCTCCTTTCTTACGAGG |
| SM1605 BamHI | CG**ggatcc**CTAGTCGCTGCGTAACATGC |

Restriction enzyme recognition sites are indicated in bold, lower case letters and mismatched nucleotides for insertion of mutations are depicted in italic, lower case letters.
